# Microsurgical training course for clinicians and scientists: a 10-year experience at the Münster University Hospital

**DOI:** 10.1186/s12909-021-02737-1

**Published:** 2021-05-24

**Authors:** Mazen A. Juratli, Felix Becker, Daniel Palmes, Sandra Stöppeler, Ralf Bahde, Linus Kebschull, Hans-Ullrich Spiegel, Jens P. Hölzen

**Affiliations:** Department of General, Visceral and Transplant Surgery, Münster University Hospital, Münster, Germany

**Keywords:** Microsurgical training, Anastomosis, Microsurgery, Vascular anastomosis, Training, Curricular training

## Abstract

**Background:**

Microsurgical techniques are an important part of clinical and experimental research. Here we present our step-by-step microsurgery training course developed at the Münster University Hospital. The goal of this course was to create a short, modular curriculum with clearly described and easy to follow working steps in accordance with the Guidelines for Training in Surgical Research in Animals by the Academy of Surgical Research.

**Methods:**

Over the course of 10 years, we conducted an annual 2.5 day (20 h) microsurgical training course with a total of 120 participants.

**Results:**

Prior to the course, 90% of the participants reported to have never performed a microanastomosis before. During the 10 years a total of 84.2% of the participants performed microanastomoses without assistance, 15% required assistance and only 0.8% failed.

**Conclusions:**

Our step-by-step microsurgery training course gives a brief overview of the didactic basics and the organization of a microsurgical training course and could serve as a guide for teaching microsurgical skills. During the 2.5-day curriculum, it was possible to teach, and for participants to subsequently perform a microsurgical anastomosis. The independent reproducibility of the learned material after the course is not yet known, therefore further investigations are necessary. With this step-by-step curriculum, we were able to conduct a successful training program, shown by the fact that each participant is able to perform microvascular anastomoses on a reproducible basis.

## Background

Microsurgical techniques are being increasingly applied in almost all surgical disciplines. Due to an increase in the number of microsurgical operations in recent times, the number of trainees in this highly specialized surgical field has continued to grow. In addition, microsurgical methods are an essential component of surgical research, ranging from central and peripheral vascular cannulation [1–3] to complex and sophisticated surgical models [4, 5], including models of organ transplantation [6, 7]. Its significance is based on the possibility of evaluating new surgical methods and materials, gaining better understanding of pathophysiology and testing new ideas and innovations [8–10]. Proper education and training benefit not only the trainees, but also the research institutions and patients.

Because microsurgery offers a broad spectrum of techniques and skills, a training program must be developed that considers demands and requirements of the different staff in hospital and research units. Young physician’s medical students performing their doctoral theses and increasing numbers of researchers must be taught thoroughly and by standardized methods. Due to amendments of laws, changes in the education of resident students and the increasing lack of physicians, there has been an increase of non-surgeons involved more and more in research.

However, many hospitals and research units have neither an educational concept nor the needed resources. They are not able to offer workplaces, tools and experienced instructors to teach surgeons, residents and researchers in microsurgical techniques. This has resulted in a growing interest in microsurgical training courses [11]. Due to the enormous range of courses currently offered, it is not easy to choose the best one. A good course requires an efficient and motivating teaching concept and is characterized by a clear curriculum with transparent tasks. Each task should be achieved within a realistic timeframe and reflect a stepwise increase in complexity. In addition, it is important to provide a positive learning environment (e.g. no distraction, proper instruments) and to provide mentored experiences and guidance in difficult situations. As important as the evaluation by a mentor is a constant self-evaluation, to build confidence in surgical skills.

The sutured vascular anastomosis holds a special position in the here described curriculum. This microsurgical technique is very frequently applicable in clinical work; it is, for example, used in the surgical research field (e.g. in organ transplantation). Di Cataldo et al. describe the fundamentals of every anastomosis to be a precise clear cut of the vessel as well as an avoidance of adventitia getting in the lumen and too much pull on the vessel stump. Other essential elements are tension free stumps without kinking of the vessel; washing out the inside of the vessels with saline solution; and the introduction of the needle, with one-stitch clean-cut needle flow perpendicularly to the vessel wall without piercing both edges of the vessel [8, 9]. Moderately placed sutures avert the danger of a stenosis in the continuous suture.

In our department at the Münster University Hospital, a related educational concept has existed for more than two decades. The idea therefore arose to offer a training course that is clear and easy to understand, not only to teach microsurgical techniques, but also the use of necessary microsurgical equipment. This article is intended to give a brief overview of the educational basics, organization and execution of hands-on courses for vascular microsurgery and also to serve as a guide for teaching microsurgical skills.

## Methods

### Animals

This study was approved by the University of Münster Ethics Committee and was carried out in compliance with the ARRIVE guidelines. All experiments involving living animals were performed in accordance with the German Animal Welfare Law and beforehand approved by the administrative authority of North Rhine-Westphalia (reference numbers A46/2002 and 81–02.05.40.18.016). Male and female Wistar rats (Charles River, Sulzfeld, Germany) weighing 150-200 g were used. All operations, applications, and investigations were carried out under intraperitoneal anesthesia with ketamine and xylonest.

### Setup of the course

The proposed curriculum is based on the data collected during the ten hands-on courses with a total of 120 participants that were conducted in our department. The course concept (teaching resources, manners, aims, equipment) and execution remained unchanged during the 10-year study period.

All participants were informed about identified analysis of registered data and informed consent of each participant was obtained. All methods were carried out in accordance with relevant guidelines, regulations and approved by a named institutional and/or licensing committee (Ethics Commission of the Westphalia-Lippe Medical Association and the Westphalian Wilhelms University). However based on the study setup, there was no need for any ethical application.

The aim of the courses was to learn essential skills in microsurgery of the peripheral blood vessels. The main goal was for each participant to be able to independently perform vascular a microanastomosis in a living rat. Microsurgical suturing of anastomoses largely accounts for the complexity of many experimental microsurgical models [8, 9]. Mastering the microanastomosis technique is therefore at the center of the training program. This skill can also be transferred to everyday clinical and experimental work.

**Fig. 1:**
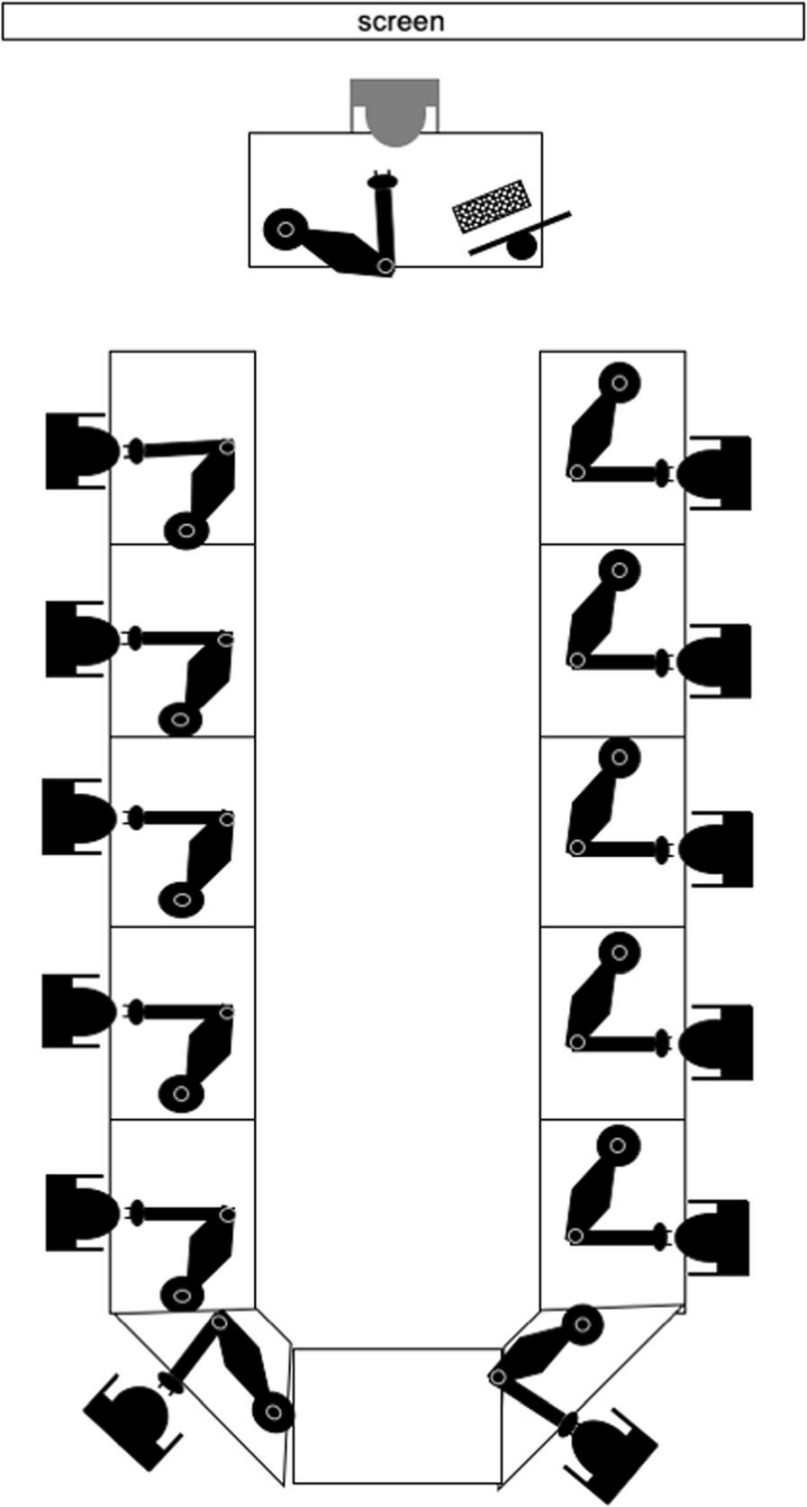
Workplace. Each participant works with a binocular microscope. Each microscope has an extra eyepiece

**Fig. 2:**
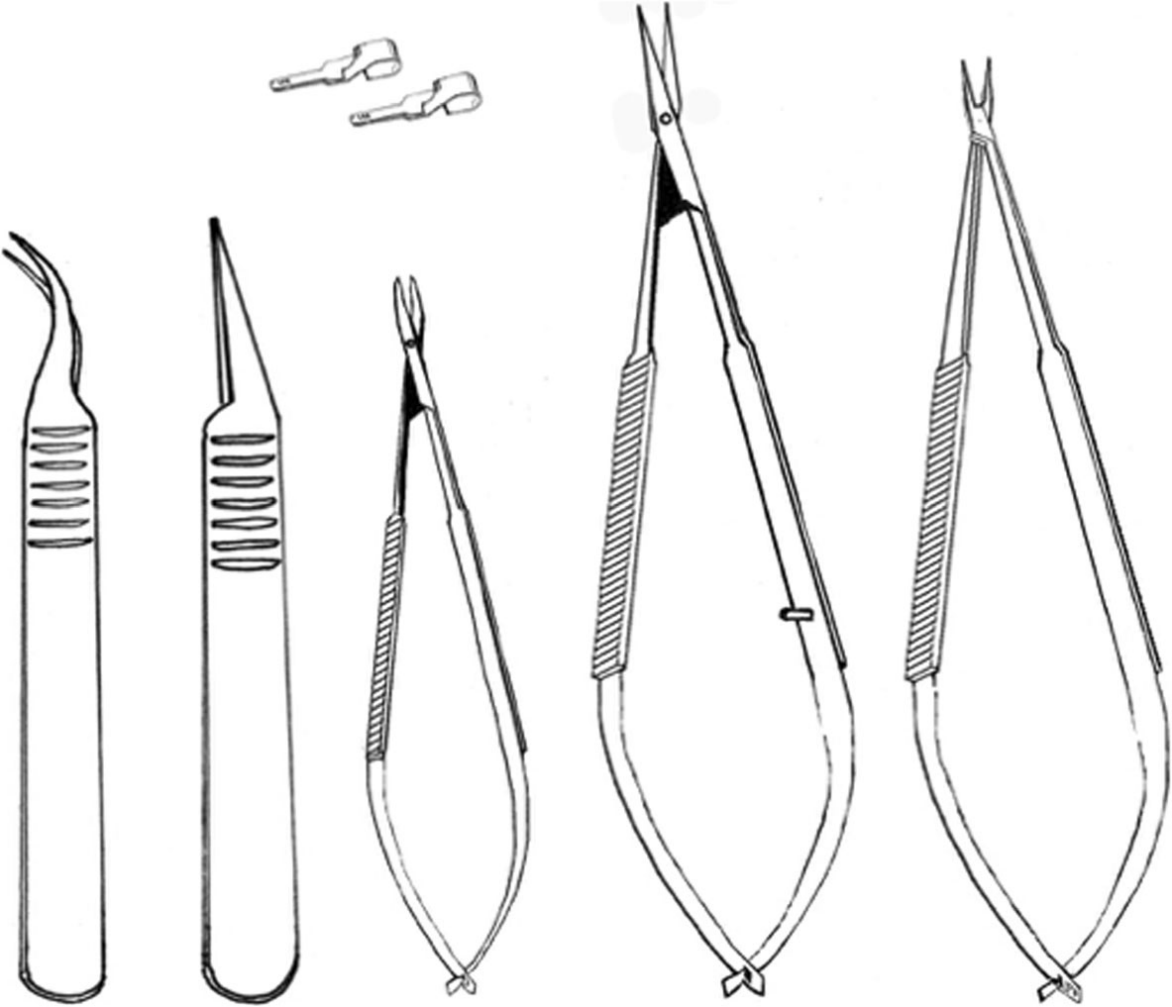
Instrument set. Straight and curved micro-forceps, straight and curved micro-scissors, micro needle holder, 2 x Biemer vessel clips

The course took place in a large conference room. There were 12 tables in U-Form and on each table there was a surgical microscope ((Zeiss® - OPMI 1-FC, Jena, Germany). In addition, everyone has a worktop with a straight surface (Fig. 1). Following microsurgical instruments were used: Straight and curved micro-forceps, straight and curved micro-scissors, micro needle holder, 4 x Biemer vessel clips in different sizes (Fig. 2). All operations, applications, and investigations were carried out under inhalatory nitrous oxide/oxygen isoflurane anesthesia (N_2_O/O_2_ = 2:1 + 1.5 vol% isoflurane). Suture materials 8–0, 9–0 and 10–0 nylon were used.

The group of participants consisted of 80% researchers and 20% surgeons. Combined theoretical and practical training, however, is also necessary; without a background of theory, it is impossible to understand the practical steps and incorporate them into a logical working algorithm. Theoretical lectures were, therefore, held in blocks. Before the first practical session, a lecture was held about optical aids and microsurgical instruments.

Each participant received 10 practical tasks with defined educational goals, also considered to be stages. These stages built upon each other in order of their difficulty. Each practice was introduced by a live video demonstration of the techniques.

To ensure optimal supervision during the practical exercises, the total number of participants for each course was limited to a maximum of 12, supported by 4 instructors, which means there was a participant-tutor ratio of 3:1. The instructors were recruited from the Department of General, Visceral and Transplant Surgery, having considerable experience in experimental microsurgical methods and in clinical applications of microsurgical techniques.

Adequate technical equipment, required for the learning environment, was supplied for participants. We limited ourselves to only the necessary number of tools needed to accomplish the tasks. The microsurgical workplace comprised a binocular operating microscope (Zeiss® - OPMI 1-FC) and the participants had the opportunity to work with their own instruments. We equipped one master microscope with a camera and connected it to a video projector [12, 13].

Simulation is a main principle of the curriculum. It can reduce the number of animal experiments and save costs. Another benefit of simulation is the hands-on training with synthetic tissues and real-time feedback, which increases the learner’s motivation [14] and successes [15]. In contrast to high fidelity simulations, which are costly, low fidelity simulations use materials and equipment that are less similar to those used clinically. They are, however, still a good teaching tool because they have the advantages of being inexpensive, easy to use, easily reproducible and easily available.

Before starting microsurgical work at the micro level, it is advisable to perform simulations at the macro level; to learn, practice and memorize the various working steps before performing them under more difficult circumstances using a microscope. As a first step, the trainee performs simulation exercises to learn how to handle tools on non-living materials such as plastics, which are easier to supply and usually less expensive. Perforated stencils or a plaster roll with a coating of latex and rubber hoses are also acceptable materials for the beginner to learn both suturing and how to perform vascular anastomoses [16–21]. In this way, the beginner.

gains an understanding of the work and can be introduced to the incorporation of the microsurgical instruments step-by-step into the basic stages of work.

All participants were given a self-evaluation prior to and after the course (Table 1).

**Table 1:** Evaluation criteria: 1: perform independently; 2: perform with little support; 3: perform with clear support; 4: only basic theoretical knowledge; 5: insufficient theoretical and practical knowledge

	1	2	3	4	5
**Handling a surgical microscope**					
**Handling a surgical instrument**					
**Handling a microsurgical instrument**					
**Handling sutures - macro**					
**Handling sutures - micro**					
**Implementation of manual and instrumental knots**					
**Carrying out knots under the microscope**					
**Performing a vascular anastomosis, diameter > 5 mm**					
**Perform a micro vascular anastomosis, diameter < 5 mm**					
**Performing a nerve suture**					

### Ten step training program

**Fig. 3:**
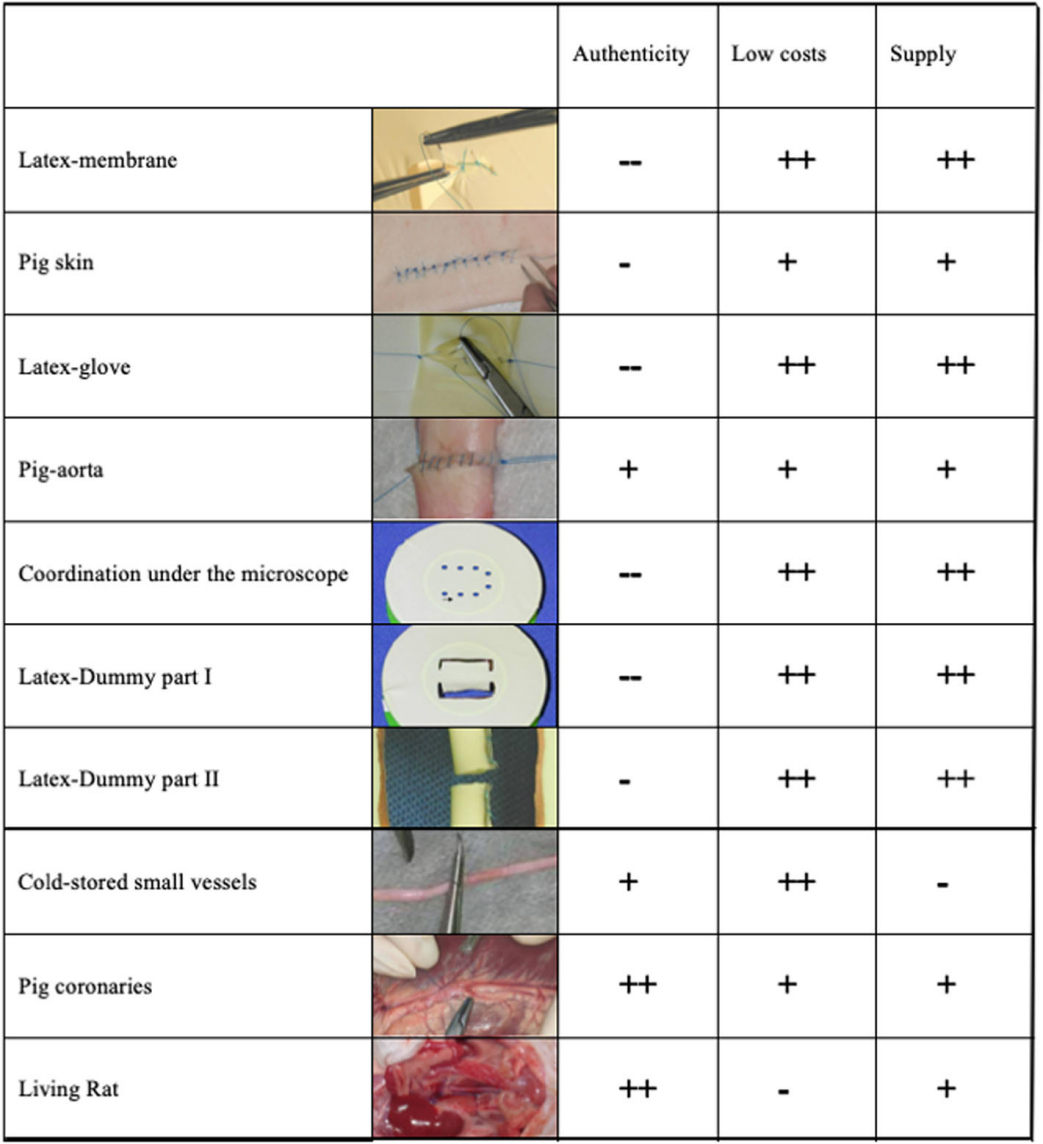
Practical exercises are divided into 10 Steps. A good simulation is characterized by a high degree of authenticity, low cost and a good supply

The aim of the program is to develop an effective, transparent curriculum that will motivate learners. Practical training is divided into ten steps. Each step, outlined below, contains formulated learning targets in order of their difficulty (Fig. 3).

#### Step 1: latex diaphragm

The educational goal in this exercise is the handling of instruments at the macro range; the rotating motion of the needle and the instrument knot. A latex glove is stretched over a petri dish and a 2 cm incision is made. Then, a single knot suture is performed (polypropylene 5–0) with a spacing interval of 5 mm, using instrument knots.

#### Step 2: pig skin

As a next step, the trainees have to learn the feeling of suturing in organic material and the meaning of the correct needle rotation. Pig skin is attached to a styrofoam board and 2 × 4 cm long incisions are performed. Then, continuous suture is carried out with polypropylene 5–0 and an interstitch distance of 5 mm.

#### Step 3: glove finger anastomosis

The educational goal for step 3 is the successful completion of an anastomosis with a continuous suture within the macro range. A latex glove is fixed on a styrofoam board and a finger (of the glove) is transected. After setting the corner sutures (2x polypropylene 5–0 leaving sutures with needle attached), a transvascular suture at the opposite posterior wall is performed in a continuous fashion using one of the corner sutures. After knotting the corner suture, in a similar way to the previous exercise, the front wall is sutured in the same way as the posterior wall. After knotting is completed, the quality of the anastomosis can be checked by trying on the glove. By this simple test, it is checked whether the posterior wall was accidentally incorporated into the front wall layer and if the nots have been tightened too firmly and thus constricted the lumen. In addition, regularity (knot to knot distance, distance between puncture and cut out) of the suture row is checked and assessed.

#### Step 4: anastomosis of a pig aorta

The educational goal of this step is the successful completion of an anastomosis with a continuous suture anastomosis in organic tissue with its intima. After setting the corner sutures, the posterior wall and anterior wall are sewn in a continuous fashion with polypropylene 6–0. To reach the posterior wall, the needle is driven inside by an outside-inside stitch before continuing the transvascular suture. After knotting is completed, the quality of the anastomosis can be assessed by a longitudinal incision of the aorta to enable inside vision to check for regularity (knot to knot distance, distance between puncture and cut out) as well as full thickness of the stiches, assessing any eventual intima dissection. This is the last macroscopic exercise and all further steps are performed using the microscope.

#### Step 5: coordination model under the microscope

This is the first exercise using the microscope. Beginning with a 0.4x magnification, participants can further advance to higher magnifications up to 2.2x. However, usually a 1.6x magnification is the optimal choice. The educational goal is the three-dimensional handling of microsurgical instruments with both hands using magnifying optical conditions. The binocular microscope has several magnifying levels. On each level, stitches are practiced in different directions on a surgical glove-based card model. This coordination model is easily prepared by stretching a latex glove over a plaster roll. The glove will then be marked with points, which are to be stitched with polypropylene 8–0.

#### Step 6: dummy - part I of II

**Fig. 4:**
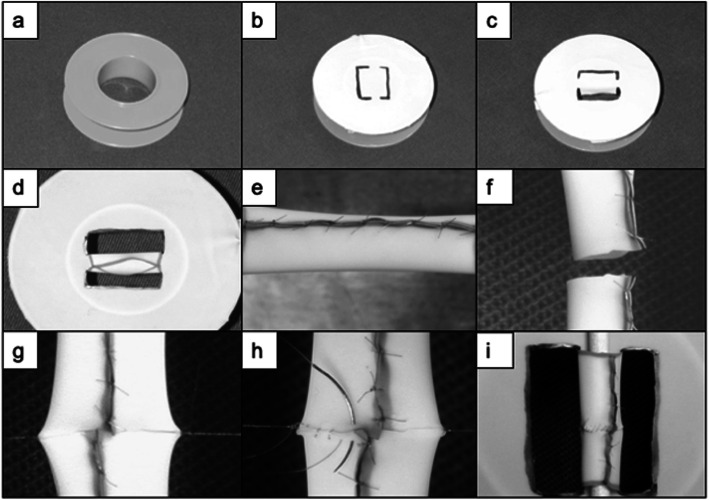
The “Dummy”. The dummy represents the perfect low fidelity simulation for microanastomosis. First, a roller is covered with latex (**a**, **b**), then a “[·]” is cut out (**c**). A tube is sewn, using single-stitch technique (**d**, **e**). This tube is cut and then sewn together, using running suture (**f**-**i**)

The objective in step 6 is to learn how to apply sutures and to tie knots using the microscope. The dummy exercise is one of the most important low fidelity simulations. It unites many skills (working in a small and narrow field of view, eye-hand coordination, use of fine instruments as well as thin sutures) using the microscope and offers the opportunity to practice cuts, sewing and knotting. The successful completion of three-dimensional work using the microscope, in different levels, as described before, is even possible. The exercise is based on a plaster roll. In order to prepare it, the roll is placed inside a surgical latex glove, and then a figure is drawn on the glove. After incision of the figure using microscissors, single knots are performed with a spacing interval of 1.5 mm, so that a tube is formed (Fig. 4a-e). After initial trials with sutures of a larger gauge, the beginner should proceed to finer and finer materials (nylon), using sutures of down to a gauge of 8–0 or 9–0.

#### Step 7: dummy - part II of II

The objective here is to sew a continuous end-to-end suture using the microscope. After step 6 has been completed, the needle is driven from outside to inside, the tube is transected at mid-point, corner sutures are performed, and posterior wall (transvascular) and front wall are sewn in a continuous fashion with 2x nylon 8–0 or 9–0 leaving sutures with needle attached (Fig. 4f-i).

#### Step 8: cold-stored small vessels

The next step is to perform microscopic exercises with organic materials. The educational goal is the successful completion of an anastomosis with a continuous suture in preserved rat aorta’. Saline-frozen segments of aorta (2 cm long) are used for this purpose [22]. After setting the corner sutures, the posterior wall and anterior wall are sewn in a continuous fashion with 2x polypropylene 8–0 leaving sutures with needle attached. The quality of the anastomosis can be assessed by longitudinal pull of the vessel.

#### Step 9: anastomosis of a pig coronary artery

Pig coronary arteries are an excellent material for training microvessel anastomoses. Pig hearts are inexpensive and easy to obtain. The aim of this part of the training program is to learn the techniques of end-to-end microsurgical vascular anastomosis with continuous sutures. Successful learning can be displayed by perfusion with colored saline [11, 23, 24]. After preparation and longitudinal pull (gentle pull on both corner stitches) of the coronary artery, corner sutures are performed and posterior wall and anterior wall are sewn in a interrupted or continuous fashion with 2x nylon 8–0 or 9–0 leaving sutures with needle attached. When interrupted stitches are used, the corner stitches are placed first, then the front wall is sutured followed by flipping of the vessel (turning the posterior wall to the front by changing the side of the corner stitches) and suturing of the posterior wall.

#### Step 10: living rat

After working with these non-living imitations, the trainees should feel confident enough to perform a vascular anastomosis on the artery of a living rat. The objective is an end-to-end anastomosis of the infrarenal aorta using continuous or interrupted suture techniques. After median laparotomy, the abdominal cavity is opened, the intestine convolute is put on the right side, and the aorta and vena cava are prepared and separated. After setting two Biemer clips, disconnection, and flushing, the corner sutures are carried out. Posterior and anterior wall are sewn in a running fashion or with single knot sutures with nylon 9–0. After opening the clips, hemorrhages have to be controlled by applying soft pressure with warm and moist gauze. In cases of major leakage, an additional single stitch (nylon 9–0) was placed. The next step is an end-to-end anastomosis of the infrahepatic vena cava. The difficulty here lies in the finer wall. The anastomosis is to be performed as described above (using either continuous or interrupted suture techniques) with nylon 9–0. Depending on the obtained skills and choice of described above, arterial anastomoses of the portal vein, the carotid artery, and the femoral artery can also be practiced during this last phase of the course. Anastomotic patency assessed in the rat is assessed visually in terms of checking for pre anastomotic dilatation, post anastomotic shrinkage and bleeding. Moreover, patency can be assessed after the animal has been euthanized by exercising the tissue, flushing it and lastly open it longitudinally.

## Results

**Fig. 5:**
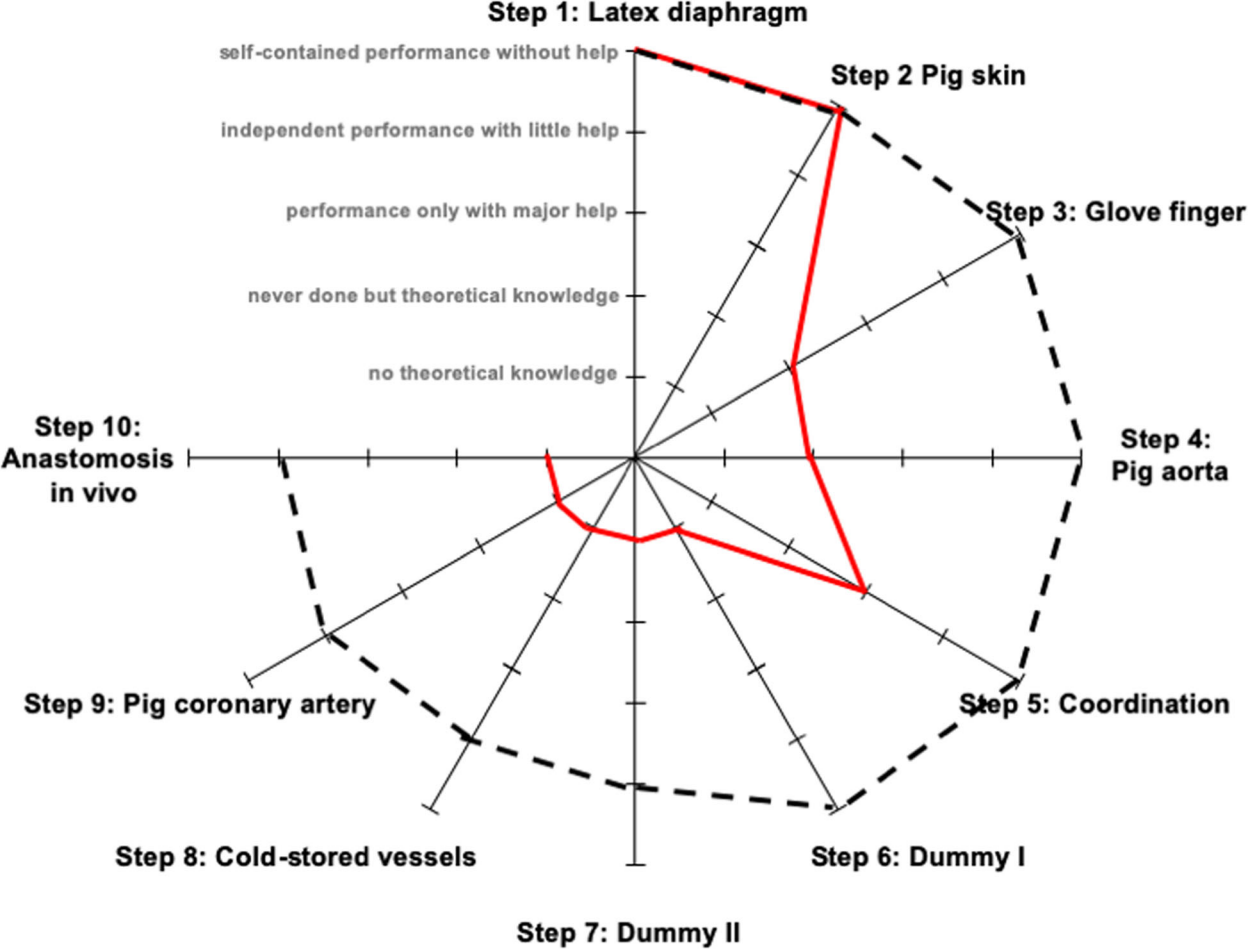
Self-assessment of participants regarding independent performance of 10 learning steps before and after the curriculum. All steps were outlined before evaluation; the participants were then interviewed the first time. At the end of the course a second self-assessment was obtained. The median is shown on the five-step-scale (“not theoretical knowledge” to “self-contained performance without help”). The red curve represents the assessment before the course. The black dashed curve is the self- assessment after the course

Almost all the participants reached the goal of performing a microanastomosis. Over the course of 10 years, 101 of the participants (84.2%) were able to complete the anastomosis without assistance, 18 participants (15%) required the assistance of an instructor, and only 1 participant (0.8%) failed and could not perform an anastomosis even with major help of an instructor. All participants were given a self-evaluation prior to and after the course (Fig. 5). The majority of the participants had experience in experimental surgery but only 30% of all participants had microsurgical knowledge.

Most participants, however, had insufficient knowledge of how an anastomosis should be sewn. This was even true regarding the exercises using the microscope. Figure 5 gives an overview of participants’ knowledge before and after the course. It shows their self-assessment regarding the independent performance of the 10 learning steps before and after the curriculum. Before the course, all participants had knowledge of macro instruments and simple sutures. From Step 3 (glove finger anastomosis), we observed a significant decline in self-assessment.

Following the course, we have seen a significant improvement in self-assessments. The majority of the participants were confident in independently performing up to step 6 of the curriculum. All other steps including step 10 were accomplished with a little help.

Prior to the course, two thirds of the participants stated that they had no theoretical or independent implementation knowledge of macroanastomosis. After the course, 78% were able to perform a macroanastomosis without any assistance. Self-evaluations in regards to the microsurgery were similar. Before the course, 90% of the participants had never performed a microanastomosis. After the course, 84% reported to be able to do a self-contained performance without (62%) or with a little help (22%).

## Discussion

Due to the broad spectrum and challenging nature of microsurgery, there is an eminent need for a structured concept of microsurgical education and skills-training. We present a successful curriculum that has been carried out by our Department of Surgery over two decades. Our department has been developing clearly defined teaching concepts for many years. A properly funded education benefits trainees as well as hospitals and university departments. The established methods can be continuously used and reproduced with the highest level of quality assurance.

In our course, we pursue three main educational principles: 1. Theory before praxis – a fundamental grasp and understanding of the theoretical material is essential for success in praxis; 2. Macroanastomosis before microanastomosis – it is important to have a full understanding and enough practice of the training steps. It is too difficult to concurrently learn a new technique and handle magnifying optical aids; and 3. Sequence of material - due to animal protection laws, hands-on-training starts with synthetic materials, followed by biological tissues. Living animals are used only after the theoretical knowledge has been gained and all hands-on-training exercises have been completed.

In general, simulation can shorten the learning curve if used well; breaking down the complexity of a learning situation for instructional benefit. It can give participants the skills and confidence necessary to move onto living animals. Simulations can save money and enhance safety and quality. They are designed to reproduce aspects of the working environment; whether limited to a single task or used for complex steps all the way to recreating an entire work environment [25]. Low fidelity simulations use materials and equipment that are less similar to those used clinically. However, they have the advantage of being inexpensive, reliable, portable, easy to use, easily reproducible and easily available. The dummy exercise in particular offers several technical options. The imitation prepared with a plaster roll and a latex glove is easy to build and very inexpensive. By creating smaller diameters of tubes/mock vessels, it is possible to suture the end-to-end anastomosis at a higher level of difficulty.

The sutured vascular anastomosis holds a special position in the curriculum. This microsurgical technique is very frequently applicable in clinical work; it is, for example, used in the surgical research field (e.g. in organ transplantation).

We intentionally created a curriculum with the final educational goal of performing an anastomosis on a living animal. Which is also an advantage over the most other offered courses. Ethical arguments against animal use are justified and therefore lead to various nonliving imitations for teaching microsurgery [14, 19, 24]. However, living animals are still needed and justified to simulate the clinical situation, once the participants have reached a high level of tissue control and security in performing vascular anastomosis [11]. Our curriculum meets the recommendations of the guidelines for training in surgical research in animals by the Academy of Surgical Research and the guidelines for veterinary care of laboratory animals [26]. Participants start with non-living imitations, where they can gain experience in the use of microscopes and microscopic instruments and techniques. This reduces the number of required animals.

This kind of studies has some limitation. There is no doubt that an organization requires enough microscopes and microsurgical instruments for everyone to use in learning new techniques. However, with the help of a master microscope including video projection, exercises can be demonstrated in detail and individual questions can be answered in a detailed way. Cooperation with outside companies might be needed in order to solve equipment issues, since the equipment is an indispensable aspect of this process. Additional limitation is the short duration of the course to have enough time to repeat the microsurgical anastomoses without mistakes. Undoubtedly, the group of participants was very diverse, which was partly due to the high proportion of non-clinical staff. The self-assessment showed us that there was a high degree of knowledge in experimental practice but very little knowledge about anastomosis. It was therefore even more impressive to see how good the results were at the end of the course. While the evaluation during the course clearly demonstrates that our course is able to teach short term skills, it would have been interesting to conduct a post-course survey to evaluate the effect of the course on the participant’s professional career. It can only be assumed that the course enabled the participants to conducting complex procedures at their home institutions.

## Conclusion

During the 2.5-day curriculum, it was possible to teach and for participants to subsequently perform a microsurgical anastomosis. The independent reproducibility of the learned material after the course is not yet known, therefore further investigations are necessary.

## Data Availability

The datasets used and/or analyzed during the current study are available from the corresponding author on reasonable request.
